# Point of Care Quantitative Assessment of Muscle Health in Older Individuals: An Investigation of Quantitative Muscle Ultrasound and Electrical Impedance Myography Techniques

**DOI:** 10.3390/geriatrics3040092

**Published:** 2018-12-16

**Authors:** Lisa D Hobson-Webb, Paul J Zwelling, Ashley N Pifer, Carrie M Killelea, Mallory S Faherty, Timothy C Sell, Amy M Pastva

**Affiliations:** 1 Duke University Department of Neurology/Neuromuscular Division, Durham, NC 27710, USA; paul.zwelling@duke.edu (P.J.Z.); ashley.pifer@duke.edu (A.N.P.); 2 Duke University Department of Orthopaedic Surgery, Durham, NC 27710, USA; carolyn.killelea@duke.edu (C.M.K.); mallory.faherty@duke.edu (M.S.F.); tcs30@duke.edu (T.C.S.); amy.pastva@duke.edu (A.M.P.); 3 Michael W. Krzyzewski Human Performance Laboratory, Durham, NC 27710, USA; 4 Duke University Claude D. Pepper Older American Independence Center Durham, NC 27710, USA

**Keywords:** elderly, aging, muscle, ultrasound, electrical impedance myography, point of care, TUG, frailty

## Abstract

*Background:* Muscle health is recognized for its critical role in the functionality and well-being of older adults. Readily accessible, reliable, and inexpensive methods of measuring muscle health are needed to advance research and clinical care. *Methods:* In this prospective, blinded study, 27 patients underwent quantitative muscle ultrasound (QMUS), standard electrical impedance myography (sEIM), and handheld electrical impedance myography (hEIM) of the anterior thigh musculature by two independent examiners. Subjects also had dual-energy X-ray absorptiometry (DEXA) scans and standardized tests of physical function and strength. Data were analyzed for intra- and inter-rater reliability, along with correlations with DEXA and physical measures. *Results:* Measures of intra- and inter-rater reliability were excellent (>0.90) for all QMUS, sEIM, and hEIM parameters except intra-rater reliability of rectus femoris echointensity (0.87–0.89). There were moderate, inverse correlations between QMUS, sEIM, and hEIM parameters and measures of knee extensor strength. Moderate to strong correlations (0.57–0.81) were noted between investigational measures and DEXA-measured fat mass. *Conclusions:* QMUS, sEIM and hEIM were highly reliable in a controlled, same-day testing protocol. Multiple correlations with measures of strength and body composition were noted for each method. Point-of-care technologies may provide an alternative means of measuring health.

## 1. Introduction

Muscle health is recognized for its critical role in the well-being and functionality of older adults [1]. Aging is associated with loss of muscle mass, known as sarcopenia, and loss of muscle quality, manifesting as replacement of healthy muscle tissue by adipose and water. Both have been associated with limitations in physical activity and function, including the performance of routine activities of daily required for independent living [2,3]. To date, muscle health assessment has primarily focused on muscle quantity; however, this assessment alone may overestimate the amount of functional muscle. Computed tomography (CT), magnetic resonance imaging (MRI), and dual energy X-ray absorptiometry (DEXA) scans are gold standard measures used to assess the quantity and quality of muscle [4]. CT has most frequently been used to assess fatty infiltration of muscle, which manifests as a reduced attenuation coefficient [5]. However, given that these scans are expensive, not readily accessible in the outpatient or community setting, or well-suited for home use, there is a need for novel means of measuring muscle health for both clinical and research purposes.

Ultrasound provides a rapid, non-invasive, portable and inexpensive means of evaluating muscle. Although new approaches, including shear wave elastography, are being investigated, measures of muscle size and signal are traditionally used. Muscle size is often expressed as thickness or cross-sectional area of the muscle, while signal is described in terms of echointensity (EI). As expected, reduced muscle size at select sites has been linked to an overall reduction in muscle mass. Studied primarily in disease states, elevated EI represents increased intramuscular fibrous composition and generally correlates with worsening muscle health [6]. Other factors that can affect EI include ultrasound system settings, aging, sex and level of conditioning, making its interpretation somewhat challenging [7,8].

A number of recent studies have explored the role of quantitative muscle ultrasound (QMUS) in the critically ill, but less is known about the role of muscle health in community-dwelling elderly individuals [9,10]. An early study of 92 elderly Japanese patients demonstrated moderate inverse correlations between EI and quadriceps strength among middle-aged and elderly individuals, independent of age or muscle thickness [11]. More recently, Mirón Mombiela et al. examined EI as biomarker of frailty in a study of patients aged 20 to 90 years [12]. The group found, similar to the aforementioned study, moderate, inverse correlations between EI and quadriceps strength. Higher EI values were also associated with greater frailty.

Electrical impedance myography (EIM) is another technology receiving recent attention for measurement of muscle health. Differing EIM devices are available, but the basic principle is that these systems measure the muscle impedance to flow of a painless, electrical current. Impedance is not very high in healthy muscle but is known to increase in conditions that impact normal muscle architecture [13]. Prior studies in amyotrophic lateral sclerosis (ALS) and Duchenne muscular dystrophy (DMD) demonstrate the promise of this technique [14,15,16]. Unlike imaging studies, EIM can be performed with little training. It is portable and hand-held devices are available. The equipment is inexpensive; some consumer-marketed devices are available for less than US $100. These advantages are appealing, but data on reliability, longitudinal change, correlation with established measures of strength, and the impact of patient positioning, hydration and many other factors are lacking.

Given the need for improved muscle health data and the advantages of QMUS and EIM, the current study was designed to investigate the feasibility of using both technologies in the community-dwelling elderly. The aims of the current study were to determine the inter-rater reliability and reproducibility of QMUS and EIM. In addition, we examined the correlations between QMUS, EIM and currently accepted measures of physical strength, physical function, and muscle mass. The findings demonstrate that both QMUS and EIM are reliable tools that correlate with functional and DEXA measures.

## 2. Materials and Methods

### 2.1. Research Cohort

The Duke Institutional Review Board approved the current study (Duke IRB Protocol 00076633). Twenty-seven subjects were recruited through use of the Pepper Center (Duke IRB Protocol 00016209)/Duke Aging Center Subject Registry (Duke IRB Pro00005016) from January to December 2017. Subjects were recruited through letters sent to individuals that had previously consented to being listed in the Duke Aging Center Subject Registry. Flyers were also posted at geriatric clinics and in physical therapy clinics around campus. Interested subjects replied to the study coordinator to learn more about the study before the informed consent process took place.

Inclusion criteria were age >65 years and the ability to provide informed consent. Those unable to provide informed consent were excluded. Patients with known active malignancy, myositis, motor neuron disease, inability to ambulate independently or taking daily steroids were also excluded as these factors can affect muscle bulk rapidly. 

Upon providing informed consent to participate in the study, subjects underwent testing (estimated time involvement 3–4 h) over 1–2 site visits. All study activities were completed within a 7-day period. QMUS and EIM were performed on the same day for all patients studied.

### 2.2. Investigational Techniques

#### 2.2.1. Quantitative Muscle Ultrasonography (QMUS)

QMUS of the right rectus femoris and vastus intermedius complex was performed at one-third the distance between the patella and anterior superior iliac spine. The subject was positioned at rest in the supine position with the knee in passive extension. Two independent examiners (LHW, PJZ) each collected three separate images at this site. LHW is a neurologist with 14 years of experience in neuromuscular ultrasound, while PJZ is an electrodiagnostic technician trained to perform muscle ultrasound for the purpose of the current study. PJZ had 2 weeks of training prior to study initiation, provided by LHW. The examiners were blinded to the results of each other’s imaging and other study results.

A Esaote MyLabSIX Ultrasound system was used, equipped with a 6–18 MHz linear array probe. Probe frequency was held at 6 MHz with constant gain, compression, and time gain compensation settings. Depth was adjusted as needed to accommodate the size of the patient imaged. Ultrasound data were digitally stored in the ultrasound system and processed off-line after each subject’s visit was complete.

Both examiners performed off-line independent, analysis of images. Subcutaneous fat thickness was calculated by measuring the distance from the skin surface to the superficial fascia of the muscles using on-screen calipers. Thickness of the rectus femoris and vastus intermedius was then measured in a similar manner using the femoral border and muscle fascia as landmarks. EI was measured by exporting the still images to Adobe Photoshop (Adobe Systems Incorporated, San Jose, CA, USA) for gray scale analysis scoring. The gray scale ranges from 0 to 255 (0 = black, 255 = white). A region of interest (ROI) was drawn as large as possible for each muscle, making effort to exclude fascial borders, bone and any artifact present in the image. As each examiner generated three measurements for each parameter recorded, the mean was calculated for thickness, while the mean, standard deviation, and median were calculated for gray scale scoring.

#### 2.2.2. Electrical Impedance Myography (EIM)—Standard Equipment (sEIM)

sEIM was performed over the right rectus femoris/vastus intermedius complex, as outlined in the QMUS section. sEIM was conducted with a device (SFB7 Impedimed, Inc., Pinkenba, Australia) previously used in the assessment of neuromuscular disease. The device provides a painless, surface alternating current over muscle with four adhesive electrodes placed across the muscle for recording. Measurements were taken over the muscles’ axial plane, which is the accepted standard. Three measures each of resistance (R) and reactance (Xc) and phase (θ) were recorded for each muscle at 50 kHz and 200 kHz then averaged for the mean value of each. The subjects had sEIM performed by two independent examiners (LHW, PJZ), creating two complete sets of measures. Neither examiner had experience with this device prior to the current study and were self-trained using the company provided instructional materials. The examiners were blinded to the results of each other’s testing and other study results.

#### 2.2.3. Electrical Impedance Myography (hEIM)-Handheld Device

A handheld, portable, commercially available fitness tracker device was used (Chisel, Skulpt, Inc., San Franscisco, CA, USA). This smart-phone sized device uses the same methods as previously described for sEIM but has incorporated fixed electrodes and provides users with both a body fat measurement for each muscle assessed, as well as a Muscle Quality (MQ) score derived from raw EIM data. The frequencies used with the handheld system are proprietary and cannot be selected or altered by the user. MQ is derived from resistance and reactance measures, but the values are not available on the commercial display. The MQ scale ranges from 0 to 100, with 100 being the best score possible. The scoring system is as follows: 0–20 Needs Work, 20–40 Good, 60–80 Fit, and 80–100 Athletic.

The hEIM electrodes on the device were moistened with water and then placed over the right rectus femoris/vastus intermedius complex for approximately 5 s, while measurements were made. The subjects had three hEIM measurements performed by two independent clinicians (LHW, PJZ). Neither examiner had experience with this device prior to the current study and were self-trained using the company provided instructional materials. The examiners were blinded to the results of each other’s testing and other study results.

#### 2.2.4. Standard Clinical Measures

Age, sex, height, and weight were recorded for each subject on the day of their first study visit.

#### 2.2.5. Lower Extremity Strength and Physical Function Testing

Quantitative testing of lower extremity strength was performed by personnel at the Duke Sports Science Institute’s Michael W. Krzyzewski Human Performance Lab, under the direction of authors (AMP, TCS, MSF, CMK). Personnel were blinded to other study results.

An isokinetic dynamometer (Biodex System 3 Multi-Joint Testing and Rehabilitation System, White Plains, NY, USA) was used to measure leg strength and data were assessed using the system’s proprietary software. The test protocol was maintained for all subjects as follows: concentric (CON) at 180°/s and isometric (ISO) at 0°/s at a ~60° knee angle. Subjects were placed in a comfortable, seated position on the Biodex System 3 chair and secured using thigh, pelvic and torso straps in order to minimize accessory body movements and isolate performance at the knee. The lateral femoral epicondyle was used as the bony landmark for aligning the axis of rotation of the knee joint with the axis of rotation of the dynamometer axis. Subjects were provided a warm-up session of three repetitions at 50% of maximum effort and three repetitions at 100% of maximum effort for both the ISO and CON tests. A one-minute rest period was provided prior to the actual testing and between the two different test modes (ISO and CON). Subjects were instructed to give maximum effort throughout the entire test. For the CON test, five maximal quadriceps contractions at 180°/s were performed. For the ISO test, the leg was flexed to 60°. Subjects completed five repetitions (5-s hold for each rep) with 10 s of rest between repetitions. The Timed Up and Go test (TUG) was included as a standardized and validated physical function task measure in older adults. The test measures the time taken to arise from a chair, walk three meters, turn around, walk back to the chair, and sit down. 

#### 2.2.6. Imaging Studies

Whole-body dual energy X-ray absorptiometry (DEXA) was performed on all subjects and considered to be the gold standard for muscle mass and fat calculations of the proximal right lower extremity (thigh). Staff radiologists blinded to other study results interpreted the DEXA scans.

### 2.3. Data Analysis

Study data were collected and managed using REDCap electronic data capture tools hosted at Duke University. REDCap (Research Electronic Data Capture) is a secure, web-based application designed to support data capture for research studies, providing (1) an intuitive interface for validated data entry; (2) audit trails for tracking data manipulation and export procedures; (3) automated export procedures for seamless data downloads to common statistical packages; and (4) procedures for importing data from external sources.

QMUS and EIM measures were analyzed for inter-rater reliability as measured by the InterClass correlation. Intra-rater reliability was also assessed. For QMUS, muscle thickness, subcutaneous fat thickness and muscle EI were examined for correlation with patient demographics, DEXA measures of muscle mass, and the results of isokinetic and isometric testing. For sEIM, the impedance measures were analyzed for correlation with the same set of clinical measures. For the hEIM, the correlation between clinical measures and both the MQ score and muscle fat percentage were assessed.

Statistical significance was set at *p* ≤ 0.05, while a trend is defined as *p* = 0.051–0.10.

## 3. Results

Twenty-seven volunteers were recruited and enrolled for the study. No participants were excluded upon contact with the study coordinator. The cohort was 70% male with mean age of 72.6 years. More detailed demographics and details on the absolute QMUS values are shown in Table 1. For each subject, all testing was completed within a 7-day window. QMUS and EIM were always performed on the same day and examiner 1 and 2’s measurements were separated by a period of at least 15 min.

### 3.1. Intra- and Inter-Rater Reliability

Intra-and inter-rater reliability data for all investigational techniques are summarized in Table 2.

#### 3.1.1. QMUS

Intra-rater reliability for subcutaneous fat thickness was excellent (*r* = 0.98, *p* < 0.0001 for examiner 1 and *r* = 0.99, *p* < 0.0001 for examiner 2). Rectus femoris thickness reliability was high for both examiners (*r* = 0.98, *p* < 0.0001). Vastus intermedius thickness had good agreement (*r* = 0.98, *p* < 0.0001 for examiner 1 and *r* = 0.99, *p* < 0.0001 for examiner 2). Intra-rater reliability for rectus femoris echointensity was high for both examiners (*r* = 0.89, *p* < 0.0001 for examiner 1 and *r* = 0.87, *p* < 0.0001 for examiner 2). For vastus intermedius echointensity, intra-rater agreement was similar (*r* = 0.93, *p* < 0.0001 for examiner 1 and *r* = 0.93, *p* < 0.0001 for examiner 2).

Inter-rater reliability for subcutaneous fat thickness was excellent (*r* = 0.99, *p* < 0.0001). Rectus femoris and vastus intermedius thickness demonstrated similar results (*r* = 0.98, *p* < 0.0001 and *r* = 0.97, *p* < 0.0001, respectively). Unexpectedly, mean muscle echointensity measures for vastus intermedius had excellent agreement (*r* = 0.96, *p* = 0.005), as did rectus femoris (*r* = 0.94, *p* < 0.0001).

No significant differences between examiners were found for any QMUS parameter.

#### 3.1.2. sEIM

Intra-rater reliability measures were extremely high for 50 kHz, which was not unexpected as all measures occurred within a one-second-measurement period that required no movement of electrodes. Examiner 1 performed well for R, Xc and θ. (*r* = 1.0, *p* < 0.0001). For examiner 2, R and Xc had extraordinary reliability (*r* = 1.0, *p* < 0.0001), with θ performing only slightly worse (*r* = 0.99, *p* < 0.0001). Examiner 1 intra-rater reliability at 200 kHz was the same for R and Xc (*r* = 1.0, *p* < 0.0001). There was slightly lower agreement for θ (*r* = 0.97, *p* < 0.0001) that appeared to be secondary to a single outlying value related to patient movement. For examiner 2, reliability was also high for R (*r* = 1.0, *p* < 0.0001), Xc (*r* = 0.99, *p* < 0.0001) and θ (*r* = 0.99, *p* < 0.0001).

Inter-rater measurements were separated by several minutes, but performed well. The 50-kHz sEIM had excellent inter-rater reliability for R (*r* = 1.0, *p* < 0.0001), Xc (*r* = 0.99, *p* < 0.0001) and θ (*r* = 0.99, *p* < 0.0001). For 200 Hz, measures were similar for R (*r* = 1.0, *p* < 0.0001), Xc (*r* = 0.99, *p* < 0.0001) and θ (*r* = 0.98, *p* < 0.0001). Please note that a single data point from subject 1, examiner 1 200 kHz Xc was excluded due to a data entry error and concerns over accuracy.

No significant differences were found between examiners for any sEIM parameter at either 50 or 200 kHz.

#### 3.1.3. hEIM

hEIM performed well on intra-rater reliability measures. Examiner 1 performed well on muscle quality (*r* = 0.99, *p* < 0.0001) and muscle fat % (*r* = 0.99, *p* < 0.0001). Examiner 2 did just as well for muscle quality (*r* = 0.99, *p* < 0.0001) and muscle fat % (*r* = 0.99, *p* < 0.0001). Inter-rater reliability for muscle quality was excellent (*r* = 0.98, *p* < 0.0001), as was muscle fat % (*r* = 0.98, *p* < 0.0001). It is not surprising that the reliability was essentially identical for the two measures, given that they are inter-related and reported simultaneously. No significant differences were found between examiners for hEIM-measured muscle quality or muscle fat %.

### 3.2. Correlations

Detailed information on all correlations between investigational measures can be found in Table 3 and Table 4. 

#### 3.2.1. QMUS

Rectus femoris and vastus intermedius thickness did not correlate with isokinetic normalized peak torque. However, there was a negative correlation with subcutaneous fat thickness (*r* = −0.50, *p* = 0.008). The same pattern was evident for isometric normalized peak torque, with an inverse correlation present for subcutaneous fat thickness (*r* = −0.56, *p* = 0.002). TUG time trended toward a correlation with rectus femoris thickness (*r* = −0.37, *p* = 0.06), but there were no correlations with vastus intermedius or subcutaneous fat thickness.

Rectus femoris thickness correlated with DEXA measured right thigh muscle mass (*r* = 0.53, *p* = 0.0045), as did vastus intermedius thickness (*r* = 0.54, *p* = 0.004). There was no correlation observed with subcutaneous fat thickness. For DEXA measured right thigh fat mass, there was a strong correlation with subcutaneous fat thickness (*r* = 0.81, *p* < 0.0001). There were no correlations between DEXA measured right thigh fat mass and muscle thickness.

Muscle echointensity measures were also analyzed. Rectus femoris echointensity did not correlate with isokinetic normalized peak torque or TUG time, but did inversely correlate with isometric normalized peak torque (*r* = −0.48, *p* = 0.01). Vastus intermedius echointensity did not correlate with isokinetic normalized peak torque, isometric peak torque or TUG time. There was a trend between rectus femoris echointensity and DEXA-measured fat mass of the right leg (*r* = 0.35, *p* = 0.07) that was not seen for vastus intermedius. Similarly, a trend toward an inverse correlation between rectus femoris echointensity and DEXA-measured muscle mass of the right thigh was present (*r* = −0.33, *p* = 0.09). In addition, there was an inverse correlation between vastus intermedius echointensity and DEXA-measured muscle mass of the right thigh (*r* = −0.52, *p* = 0.006)

#### 3.2.2. sEIM

At 50 kHz, moderate negative correlations were noted between isometric normalized peak torque of knee extension and R (*r* = −0.46, *p* = 0.016) and phase (*r* = −0.45, *p* = 0.02), but not for Xc. No correlations were observed between the 50 kHz measures and isokinetic normalized peak torque of knee extension. No significant correlations were found between the 50 kHz measures and TUG time, although a trend was seen for θ (*r* = 0.35, *p* = 0.07).

For DEXA measured right leg muscle mass and 50 kHz measures, there was a trend toward a negative correlation with θ (*r* = −0.37, *p* = 0.06), but not for R or Xc. For right leg fat mass, there were correlations with R (*r* = 0.65, *p* = 0.0003) and θ (*r* = 0.57, *p* = 0.002), but not Xc.

At 200 kHz, negative correlations were again noted between isometric normalized peak torque of knee extension and R (*r*=−0.57, *p* = 0.01), as well as θ (*r* = −0.54, *p* = 0.01), but not Xc. There were also negative correlations with isokinetic normalized peak torque of knee extension and R (*r* = −0.53, *p* = 0.01), as well as θ (*r* = −0.51, *p* = 0.02). There was no correlation between Xc and isokinetic normalized peak torque of the anterior quadriceps. For TUG time, there was a positive correlation with R (*r* = 0.45, *p* = 0.04) and θ (*r* = 0.49, *p* = 0.02), but not Xc.

For DEXA measured right leg muscle mass and 200 kHz measures, there was a moderate negative correlation with Xc (*r* = −0.46, *p* = 0.016). There was a trend toward a negative correlation with R (*r* = −0.26, *p* = 0.06) and θ (*r* = −0.34, *p* = 0.09). For right leg fat mass, there were correlations with R (*r* = 0.70, *p* < 0.0001) and θ (*r* = 0.72, *p* < 0.001), but not Xc.

Use of the 50/200 kHz phase ratio negated all correlations, while a 200/50 kHz θ ratio gave nearly identical results to the 50 and 200 kHz measures for correlation with functional testing (Table 3) but performed worse for correlation with DEXA-estimated thigh fat and muscle mass.

Based upon these observations, the 200 kHz setting correlated best with measures of physical function and body composition.

#### 3.2.3. hEIM

There was no correlation between isokinetic normalized peak torque of the quadriceps and either MQ or muscle fat %. For isometric normalized peak torque, there was an inverse correlation with muscle fat % (*r* = −0.49, *p* = 0.009). TUG time did not correlate with either parameter.

MQ did not correlate with DEXA-measured muscle mass of the right thigh but had a strong inverse correlation with fat mass (*r* = −0.72, *p* < 0.0001). Muscle fat % had a strong correlation with DEXA-measured fat mass of the thigh (*r* = 0.74, *p* < 0.0001) and a weaker inverse correlation with muscle mass (*r* = −0.38, *p* = 0.0492).

#### 3.2.4. DEXA Correlation with Strength and Functional Measures

DEXA-measured muscle mass did not correlate with isometric normalized peak torque, isokinetic normalized peak torque or TUG time. DEXA-measured thigh fat mass had moderate, negative correlations with both isometric (*r* = −0.52, *p* = 0.005) and isokinetic (*r* = −0.39, *p* = 0.04) normalized peak torque, but not TUG time.

Of little surprise, isometric and isokinetic normalized peak torque correlated with TUG time (*r* = −0.61, *p* = 0.001; *r* = −0.37, *p* = 0.01).

## 4. Discussion

In this pilot study, we aimed to determine if QMUS, sEIM or hEIM would be feasible tools for assessing muscle health and function in older adults. Validating each of these techniques first required an assessment of intra- and inter-rater reliability, followed by analysis of correlations with accepted measures of muscle mass, strength, and physical function. The results show that each method was reliable within and between examiners after minimal training and correlated with both DEXA and functional measures. Our study was unique in that it examined intra- and inter-rater agreement and included a mixed population of community dwelling older adults. The population consisted of both men and women; those with chronic medical conditions were not excluded. In the following sections, the results of each investigational technique are analyzed separately and compared with prior literature.

### 4.1. QMUS

The QMUS portion of our study was unique in that it examined intra- and inter-rater agreement in a mixed population of community dwelling older adults. Subcutaneous fat thickness was also measured, which has not been a focus of prior publications. Additionally, our study compared QMUS results to standard physical rehabilitation-administered strength testing, functional task measures and DEXA scans.

The intra-rater and inter-rater reliability in the current study were excellent for all tissue thickness measures (0.97–0.99). These are very similar to a recent study examining reliability for rectus femoris measurements [17]. Multiple other publications have reported similar results for anterior thigh imaging [18,19,20,21]. The intra- and inter-rater agreement for EI in the current study (0.87–0.96) aligned with previous publications. For example, Ishida et al. found an inter-rater reliability of 0.96 for EI of the rectus femoris, while we found a value of 0.94 [17]. Other recent studies have found similar values. These findings provide reassurance that QMUS measures are accurate and repeatable between examinations, as long as the imaging is performed according to a standardized protocol. 

In comparing the current QMUS with prior publications, our findings align well with the previously mentioned Fukumoto study of 92 elderly women in regards to patient age, muscle thickness, and EI [11]. This is encouraging when considering the possibility of generalizing results, as the two studies were performed in different patient populations and with different ultrasound systems. The correlations noted between QMUS parameters and strength measurements are also similar.

Lopez et al. examined the relationship between quadriceps echointensity and functional measures in 50 healthy, elderly men [22]. The quadriceps were considered as a group for the purpose of analyzing EI and the mean value was 69.78 ± 11.4, different than seen in the current study (94.16 ± 20 for rectus femoris and 62.6 ± 32 for vastus intermedius). This may reflect the inclusion of only healthy patients, a younger population, lower BMI, the averaging of all quadriceps musculature in calculating the EI and differences in ultrasound systems. Despite the differences in absolute values, the authors found similar, moderate inverse correlations between EI and strength [20].

Similar to other studies [12,22,23], we found that EI correlated better with strength measures than did muscle thickness. In their discussion, Lopez et al. note that the presence of adipose tissue might be more relevant to age-related reductions in strength than the loss of muscle mass alone. Further supporting this hypothesis is our finding that subcutaneous fat thickness had moderate, inverse correlations with muscle strength (*r* = −0.50 to −0.56). This QMUS finding is felt to be accurate, given the strong correlation (*r* = 0.81) with the gold standard DEXA measurement of right thigh fat mass. As QMUS measured subcutaneous fat thickness is much more easily obtained than DEXA measures, QMUS may provide an excellent clinical and research tool for monitoring body fat composition.

### 4.2. sEIM

sEIM performed extremely well in parameters of intra- and inter-rater reliability (range 0.97–1.0). This was not unexpected given the short interval between trials, the easily accessible muscle chosen for analysis and the standardized protocol. Over the years, different sEIM devices have been assessed, some with fixed electrodes and some using the disposable electrode method described here. A fixed electrode array would be expected to reduce error and improve results.

Both 50 and 200 kHz frequencies were tested in the current study. Most early work focused on use of the 50 kHz frequency in sarcopenia [13]. Aaron et al. 2006 found aging to be associated with reductions in muscle impedance, specifically, the 50-kHz phase, in a cross-sectional study of 100 people [24]. This was most pronounced in men and those over age 60, but only 4 people older than 75 years were included in the study. A follow-up study demonstrated lower 50 kHz reactance, not phase, with aging. Again, men seemed to suffer more decline than women [25].

In our study, moderate, inverse correlations were found between isometric normalized peak torque and both resistance and phase at 50 and 200 kHz (Table 3). The relationship was somewhat stronger for 200 kHz measures, which also displayed similar correlations with normalized isokinetic peak torque. Only the 200 kHz measures had a moderate correlation with TUG time, although a weaker trend was observed for 50 kHz phase.

Good correlations were found between both 50 and 200 kHz resistance and phase with DEXA measured fat mass (Table 4). Again, 200 kHz measures performed better. There were trends toward correlation with muscle mass, but only 200 kHz fat mass reached statistical significance.

### 4.3. hEIM

To the best of our knowledge, there is only one other published study on the direct-to-consumer hEIM device used here [26]. The purpose of testing the device was to determine if it could be deployed in homes and possibly used by patients and research subjects for longitudinal monitoring of muscle health. No modifications were made to the system and there were no attempts to extract and analyze raw data. Intra-and inter-rater reliability was excellent (0.98–0.99) for muscle quality and % fat as measured by the device. This indicates that with minimal training, the measures are reliable for use in an outpatient clinical setting, warranting larger trials.

hEIM % fat had a moderate inverse correlation with isometric normalized peak torque, but muscle quality’s correlation with this measure did not reach clinical significance. There was a strong correlation between hEIM measured % fat and DEXA measured right thigh fat mass (0.74) and a weaker correlation with the TUG time (−0.38). Again, this suggests that increased body and muscle fat have a more profound negative impact on function than muscle mass alone.

The findings here are similar to that found by McLester et al., who studied a population of healthy young adults (mean age 24–25 years). Their study found high agreement between examiners and also found hEIM to be a viable alternative for measuring body fat % [24].

### 4.4. Study Limitations

The current study is not without limitations and poses new questions. Methodologically, the frequencies used by the hEIM were not available, so a direct comparison between the sEIM and hEIM devices could not be performed. This pilot study had a small sample size (*n* = 27), but a larger cohort would be more likely to strengthen the correlations and trends seen here rather than weaken them. Using multiple muscles, as opposed to a single muscle may alter observed correlations as well. In addition, the study was not powered to detect differences between men and women, which may have been missed in this group. Furthermore, the extremely high reliability seen between examiners is likely related to the strict training provided and the fact that all investigative measures were performed on the same day.

Additional studies are needed to determine the reliability of electrical impedance myography measures over multiple visits, although data by Geisbush et al. suggest high reliability when electrical impedance measurements are separated by 3–7 days [27]. Muscle ultrasound has also demonstrated high reliability when performed on separate days [28]. The effects of hydration, changes in skin impedance and activity prior to testing may affect reliability and should be addressed in future projects. As a final note, the hEIM device was not deployed into subjects’ homes to see if they were able to use the device with ease. Future studies will require a lapse of days to weeks prior to determining the real-world reliability of QMUS, sEIM, and hEIM.

## 5. Conclusions

QMUS, sEIM and hEIM all demonstrated excellent intra- and inter-rater reliability in a controlled, same-day testing protocol. Furthermore, multiple correlations with measures of muscle strength and body composition were noted for each method. Of particular interest is the finding that in older adults, the quality of muscle (i.e., lower % muscle fat) had stronger correlations with strength and function than muscle mass alone. This was true for QMUS, sEIM, and hEIM parameters. DEXA measures of lower extremity fat and muscle mass did not outperform ultrasound or EIM in this regard, suggesting that point-of-care, non-radiating inexpensive technologies may one day replace it as the gold standard.

The current findings require replication and further analysis in larger studies but suggest that interventions to reduce body fat may improve the health of elderly individuals.

## Figures and Tables

**Table 1:** Subject demographics and group quantitative muscle ultrasound (QMUS) results.

**Male/Female**	19 (70%)/8 (30%)
**Age (years)**	72.6 ± 5 (range 65–82 years)
**Height (cm)**	172.2 ± 11 (range 152–200 cm)
**Weight (kg)**	83.3 kg ± 19 (range 52–131 kg)
**BMI (kg/m^2^)**	28.1
**Ultrasound Measurements**	
**Fat thickness (mm)**	8.8 ± 7 (range 0.6–23.5)
**RF thickness (mm)**	13.7 ± 6 (range 1.2–23.5)
**VI thickness (mm)**	13.2 ± 6 (range 0.9–22.7)
**Echointensity RF**	94.16 ± 20 (range 68.8–167.5)
**VI**	62.6 ± 32 (range 10.3–121.2)

**Table 2:** Intra- and inter-rater reliability.

Test	Intra-Rater		Inter-Rater
	Examiner 1	Examiner 2	
**QMUS**			
SQ thickness	0.98 *	0.99 *	0.99 *
RF thickness	0.98 *	0.98 *	0.98 *
VI thickness	0.98 *	0.99 *	0.97 *
RF echointensity	0.89 *	0.87 *	0.94 *
VI echointensity	0.93 *	0.93 *	0.96 **
**sEIM**			
50 kHz R	1.0 *	1.0 *	1.0 *
50 kHz Xc	1.0 *	1.0 *	0.99 *
50 kHz θ	1.0 *	0.99 *	0.99 *
200 kHz R	1.0 *	1.0 *	1.0 *
200 kHz Xc	1.0 *	0.99 *	0.99 *
200 kHz θ	0.97 *	0.99 *	0.98 *
**hEIM**			
Fat %	0.99 *	0.99 *	0.98 *
Muscle Quality	0.99 *	0.99 *	0.98 *

hEIM: handheld electrical impedance myography; Hz: hertz; QMUS: quantitative muscle ultrasound; R: resistance; RF: rectus femoris; sEIM: standard electrical impedance myography; SQ: subcutaneous tissue; VI: vastus intermedius; Xc: reactance; θ = phase; *: *p* < 0.0001; **: *p* = 0.005.

**Table 3:** Correlations between test results and functional measures.

Test	Isometric Normalized Peak Torque	Isokinetic Normalized Peak Torque	Timed Up and Go (TUG)
**QMUS**			
SQ thickness	**−0.56, *p* = 0.002**	**−0.50, *p* = 0.08**	---
RF thickness	---	---	−*0.37, p = 0.06*
VI thickness	---	---	---
RF echointensity	**−0.48, *p* = 0.01**	---	---
VI echointensity	---	---	---
**sEIM**			
50 kHz R	**−0.46, *p* = 0.016**	---	---
50 kHz Xc	---	---	---
50 kHz θ	**−0.45, *p* = 0.02**	---	*0.35*, *p = 0.07*
200 kHz R	**−0.57, p = 0.01**	**−0.53, *p* = 0.01**	**0.45, *p* = 0.04**
200 kHz Xc	---	---	---
200 kHz θ	**−0.54, *p* = 0.01**	**−0.51, *p* = 0.02**	**0.49, *p* = 0.02**
50/200 kHz θ Ratio	---	---	---
200/50 kHz Phase Ratio	**0.50, *p* = 0.01**	**0.44, *p* = 0.027**	---
**hEIM**			
Fat %	**−0.49, *p* = 0.009**	---	---
Muscle Quality	---	---	---
**DEXA**			
Thigh muscle mass	---	---	---
Thigh fat mass	**−0.52, *p* = 0.005**	**−0.39, *p* = 0.04**	---

hEIM: handheld electrical impedance myography; Hz: hertz; QMUS: quantitative muscle ultrasound; R: resistance; RF: rectus femoris; sEIM: standard electrical impedance myography; SQ: subcutaneous tissue; VI: vastus intermedius; Xc: reactance; θ: phase; ---: no significant correlations observed.

**Table 4:** Correlations between investigational tests and DEXA results.

Test	DEXA-Measured Right Thigh Fat Mass	DEXA-Measured Right Thigh Muscle Mass
**QMUS**		
SQ thickness	**0.81, *p* < 0.0001**	---
RF thickness	---	**0.53, *p* = 0.0045**
VI thickness	---	**0.54, *p* = 0.004**
RF echointensity	*0.35, p = 0.07*	−*0.33*, *p = 0.09*
VI echointensity	---	**−0.52, *p* = 0.006**
**sEIM**		
50 kHz R	**0.65, *p* = 0.0003**	---
50 kHz Xc	---	---
50 kHz θ	**0.57, *p* = 0.002**	*−0.37*, *p = 0.06*
200 kHz R	**0.70, *p* < 0.0001**	−*0.26*, *p = 0.06*
200 kHz Xc	---	**−0.46, *p* = 0.016**
200 kHz θ	**0.72, *p* < 0.001**	*−0.34*, *p = 0.09*
50/200 kHz θ Ratio	---	---
200/50 kHz θ Ratio	**−0.41, *p* = 0.041**	---
**hEIM**		
Fat %	**0.74, *p* < 0.0001**	**−0.38, *p* = 0.0492**
Muscle Quality	**−0.72, *p* < 0.0001**	---

hEIM: handheld electrical impedance myography; Hz: hertz; QMUS: quantitative muscle ultrasound; R: resistance; RF: rectus femoris; sEIM: standard electrical impedance myography; SQ: subcutaneous tissue; VI: vastus intermedius; Xc: capacitance; θ; phase; ---: no significant correlations observed.
